# Isolation and Characterization of a Novel *Salmonella* Phage vB_SalP_TR2

**DOI:** 10.3389/fmicb.2021.664810

**Published:** 2021-06-21

**Authors:** Yuting Shang, Qifan Sun, Hanfang Chen, Qingping Wu, Moutong Chen, Shuanghong Yang, Mingzhu Du, Fei Zha, Qinghua Ye, Jumei Zhang

**Affiliations:** ^1^Guangdong Provincial Key Laboratory of Microbial Culture Collection and Application, State Key Laboratory of Applied Microbiology Southern China, Institute of Microbiology, Guangdong Academy of Sciences, Guangzhou, China; ^2^State Key Laboratory of Food Science and Technology, National Engineering Research Center for Functional Foods, Synergetic Innovation Center of Food Safety, Joint International Research Laboratory on Food Safety, School of Food Science and Technology, Jiangnan University, Wuxi, China; ^3^School of Chemical Engineering and Light Industry, Guangdong University of Technology, Guangzhou, China

**Keywords:** bacteriophage, *Salmonella*, biological characteristic, genomic analysis, biocontrol

## Abstract

*Salmonella* is a widely distributed foodborne pathogen. The use of *Salmonella* phages as biocontrol agents has recently gained significant interest. Because the *Salmonella* genus has high diversity, efforts are necessary to identify lytic *Salmonella* phages focusing on different serovars. Here, five *Salmonella* phages were isolated from soil samples, and vB_SalP_TR2 was selected as a novel phage with high lytic potential against the host *Salmonella* serovar Albany, as well as other tested serovars, including Corvallis, Newport, Kottbus, and Istanbul. Morphological analyses demonstrated that phage vB_SalP_TR2 belongs to the *Podoviridae* family, with an icosahedral head (62 ± 0.5 nm in diameter and 60 ± 1 nm in length) and a short tail (35 ± 1 nm in length). The latent period and burst size of phage vB_SalP_TR2 was 15 min and 211 PFU/cell, respectively. It contained a linear dsDNA of 71,453 bp, and G + C content was 40.64%. Among 96 putative open reading frames detected, only 35 gene products were found in database searches, with no virulence or antibiotic resistance genes being identified. As a biological control agent, phage vB_SalP_TR2 exhibited a high temperature and pH tolerance. *In vitro*, it lysed most *S.* Albany after 24 h at 37°C with multiplicities of infection of 0.0001, 0.001, 0.01, 0.1, 1, 10, and 100. In food matrices (milk and chicken meat), treatment with phage vB_SalP_TR2 also reduced the number of *S.* Albany compared with that in controls. These findings highlighted phage vB_SalP_TR2 as a potential antibacterial agent for the control of *Salmonella* in food samples.

## Introduction

*Salmonella* is one of the major foodborne pathogens that affect global food producers and public health systems (Besser, 2018). To date, up to 2,600 serovars of *Salmonella* have been identified (Grimont and Weill, 2007). Most human infection cases are known to occur through cross-contamination during food preparation or from the consumption of raw or undercooked food, including poultry, beef, chicken, milk, fruits, and vegetables (Carrasco et al., 2012). Although a variety of methods [including physical (irradiation, autoclaved sterilization, and ozone), chemical (chlorine disinfectant and trisodium phosphate), and biological (plant extracts)] have been used to control the spread of *Salmonella* in foods (Alvarez-Ordóñez et al., 2011; Bajpai et al., 2012; Chen et al., 2012; Grant and Parveen, 2017; Mattioli et al., 2020), decontamination of *Salmonella* still presents challenges owing to several weaknesses of conventional methods. For example, irradiation reportedly leads to lipid oxidation, color changes, and loss of nutrients (Ravindran and Jaiswal, 2019), whereas chlorine disinfectants have been linked to carcinogenic compound production (Meireles et al., 2016).

Bacteriophages (phages) have been recognized as potential antibacterial agents in food samples (LeLièvre et al., 2019; García-Anaya et al., 2020). Owing to a number of attributes, such as their ability to infect bacteria with host specificity, self-replication, and abundance in the environment, phages have gradually attracted the interest of researchers (Harada et al., 2018; Keen and Dantas, 2018). Felix O1 is the earliest *Salmonella* phage to be identified, in the 1930s; it was isolated by Felix and Callow in the original scheme for the identification of *Salmonella typhi* (Felix and Callow, 1943). The whole-genome sequencing was completed in 2000, and the somatic receptor for this phage is lipopolysaccharide (Whichard et al., 2010). It has also been proposed as a therapeutic or decontaminating agent (Whichard et al., 2003; Radford et al., 2017). Since then, many studies have reported the isolation and successful application of phages for *Salmonella* control (Tang et al., 2019; Abhisingha et al., 2020; Li et al., 2020; Phothaworn et al., 2020). *Salmonella* serotyping is important for disease control and prevention. Different *Salmonella* serovars have different host ranges and virulence traits (Fierer and Guiney, 2001). Although *Salmonella enteritidis* and *Salmonella typhimurium* are among the most prevalent serotypes, in recent years, other serovars (previously uncommon) have emerged. For example, Lin et al. (2020) identified 156 *Salmonella* isolates, and among them, *S.* Albany (41.7%), *S.* Schwarzengrund (20.5%), *S.* Kentucky (12.8%), and *S.* Tennessee (5.1%) are the most commonly isolated serovars. *S.* Newport (56.8%) was the preponderant serovar in samples collected from 10 irrigation ponds in produce farms over a 2-year period (Li et al., 2014); furthermore, *S.* Corvallis exhibits resistance to a wide range of antibiotics (Ma et al., 2020). The host range for the vast majority of *Salmonella* phages has been reported to include *S. enteritidis* or *S. typhimurium* (Majtanova et al., 2011; Petsong et al., 2019). Only a few phages have been shown to lyse other minor *Salmonella* serovars. Furthermore, it has been postulated that due to their defense mechanisms and the high diversity of *Salmonella*, phages isolated from one country might not have the ability to lyse bacteria in other regions (Hagens and Loessner, 2009); therefore, continuously providing new phages that focus on different serovars and possess a high lytic capacity is required for practical application.

The objectives of this study were to isolate and characterize phages with infectivity against *Salmonella* serovars for a potential use as biocontrol agents. As a result, we isolated five phages from soil samples. The host range of the isolated phages was tested, and phage vB_SalP_TR2 was selected for further study as a novel phage with a high lytic potential against the *Salmonella* serovars Albany, Corvallis, Newport, Kottbus, and Istanbul. We determined the biological characteristics of phage vB_SalP_TR2, including its morphology, titer, one-step growth curve, thermal and pH stability, and comprehensively analyzed its whole genome. Further application experiments showed that this phage reduced the population of *S.* Albany in milk and chicken meat. Therefore, phage vB_SalP_TR2 could be used as a potential *Salmonella* biocontrol agent in food production.

## Materials and Methods

### Bacterial Strains and Their Culture Conditions

In total, 48 *Salmonella* strains, including 24 common serovars, were used in this study (Table 1). Of these strains, standard strains were purchased from the American Type Culture Collection (ATCC, Gaithersburg, MD, United States) and the National Centre for Medical Culture Collections (CMCC, Beijing, China), whereas the others were isolated from food sources in our laboratory. All strains were preserved in 25% glycerol (v/v) at −80°C and revived in Luria–Bertani (LB) medium at 37°C overnight. Each *Salmonella* serovar was determined using the slide agglutination test according to ISO 6579-1 (Mooijman et al., 2019).

**TABLE 1:** Host range analysis of *Salmonella* phage vB_SalP_TR2.

**Species**	**Strain ID**	**Source**	**Lytic activity**
***Salmonella* serovar**			
*S. enteritidis*	CMCC(B) 50335	Reference strain	–
*S. enteritidis*	FSCC(I) 21501369	Chicken meat	–
*S. typhimurium*	ATCC 14028	Reference strain	–
*S. typhimurium*	FSCC(I) 21501363	Duck	–
*S.* Derby	FSCC(I) 215748	Chicken meat	–
*S.* Derby	FSCC(I) 21501346	Pork	–
*S.* Indiana	FSCC(I) 215463	Pork	–
*S.* Indiana	FSCC(I) 21501417	Chicken meat	–
*S.* Agona	FSCC(I) 215781	Chicken meat	–
*S.* Agona	FSCC(I) 21501507	Chicken meat	–
*S.* Stanley	FSCC(I) 215253	Mushroom	–
*S.* Stanley	FSCC(I) 215547	Lettuce	–
*S.* Lomita	FSCC(I) 215507	Prawn	–
*S.* Lomita	FSCC(I) 215508	Shrimp	–
*S.* Braenderup	FSCC(I) 215068	Coriander	–
*S.* Braenderup	FSCC(I) 215088	Fish	–
*S.* Thompson	CMCC(B) 20023	Reference strain	–
*S.* Thompson	FSCC(I) 215990	Cucumber	–
*S.* Rissen	FSCC(I) 215264	Lettuce	–
*S.* Rissen	FSCC(I) 21501485	Pork	–
*S. infantis*	FSCC(I) 215071	Duck	–
*S. infantis*	FSCC(I) 21501044	Chicken meat	–
*S.* Potsdam	FSCC(I) 215373	Fish	–
*S.* Potsdam	FSCC(I) 215424	Duck	–
*S.* Corvallis	FSCC(I) 215728	Chicken meat	+
*S.* Corvallis	FSCC(I) 215476	Mutton	+
*S.* Newport	FSCC(I) 215184	Prawn	+
*S.* Newport	FSCC(I) 215197	Chicken meat	+
*S.* Albany	FSCC(I) 215783	Duck	+
*S.* Albany	FSCC(I) 215096	Lettuce	+
*S.* Kottbus	FSCC(I) 215702	Fish	+
*S.* Kottbus	FSCC(I) 215198	Chicken meat	+
*S.* Istanbul	FSCC(I) 215268	Chicken meat	+
*S.* Istanbul	FSCC(I) 215765	Chicken meat	+
*S.* Tallahassee	FSCC(I) 215920	Chicken meat	–
*S.* Tallahassee	FSCC(I) 215924	Chicken meat	–
*S.* London	FSCC(I) 215078	Fish	–
*S.* London	FSCC(I) 21501497	Pigeon	–
*S.* Meleagridis	FSCC(I) 215743	Duck	–
*S.* Meleagridis	FSCC(I) 21501400	Pork	–
*S.* Senftenberg	FSCC(I) 215747	Chicken meat	–
*S.* Senftenberg	FSCC(I) 21501419	Beef	–
*S.* Weltevreden	FSCC(I) 215077	Pork	–
*S.* Weltevreden	FSCC(I) 215782	Chicken meat	–
*S.* Pomona	FSCC(I) 215279	Fish	–
*S.* Pomona	FSCC(I) 215095	Fish	–
*S.* Wandsworth	FSCC(I) 215066	Fish	–
*S.* Wandsworth	FSCC(I) 215371	Beef	–

### Isolation and Propagation of Phages

*Salmonella* phages were isolated from 25 soil samples from the public parks of Guangdong through the standard spot and double-layer agar method with some modifications (Twest and Kropinski, 2009). In brief, 5 g of each sample was mixed into 5 L of saline magnesium (SM) buffer, maintaining it overnight. It was centrifuged at 5,000 × *g* for 10 min, and the phage-containing supernatant was collected through a 0.45-μm membrane. Solid MgSO_4_ was added to the filtrate with a final concentration of 50 mM and filtered through a 0.22-μm membrane. After filtration, the membrane was cut into pieces and placed into 50 ml of elution solution [3% (w/v) beef extract, 3% (v/v) Tween 80, and 50 mM NaCl] and subjected to ultrasound treatment (200 W, 40 kHz) with 10 min for complete elution (Yang et al., 2020; Zeng et al., 2021).

For the phage enrichment step, 2 ml of the 0.22-μm-filtered phage concentrate was mixed with 40 μl of logarithmic growth phase host bacteria (24 *Salmonella* serovars in Table 1) and 2 × LB medium (2 mM CaCl_2_), followed by incubation at 37°C for 24 h. The enrichment culture was passed through a 0.45-μm syringe filter to obtain the phage suspension. The process of the enrichment step was repeated at least three times to achieve a high titer of phages. The existence of phages, whether in suspension, was confirmed using the spot assay. The purification of phages was performed using the double-layer agar method. The suspension was serially diluted with SM buffer, mixed with 100 μl of logarithmic growth phase host bacteria and 5 ml of soft-top agar [LB containing 0.4% (w/v) agar], and then poured onto solid bottom agar [LB containing 1.5% (w/v) agar]. After incubation at 37°C for 5–8 h, a single plaque was selected and resuspended in SM buffer. This purification process was repeated five times, and purified phages were preserved in LB medium supplemented with 25% glycerol (v/v) at −80°C for further use.

### Characterization of Phage vB_SalP_TR2

#### Host Range Determination

The host range of these isolated phages was determined against 24 different serovars of *Salmonella* strains using spot testing with some modifications (Kutter, 2009). A 10-μl phage stock solution (∼10^10^ PFU/ml) was spotted on the top of each bacterial lawn. After incubation at 37°C overnight, plates were observed for the presence of plaques. If the plates formed clear plaques, it was regarded as the lysis of bacteria by phages (+), whereas no clear plaque formation was regarded as no lysis (−). Every test was conducted in triplicate.

#### Transmission Electron Microscopy

Phage morphology was observed using transmission electron microscopy (TEM). Isopycnic centrifugation was performed using the cesium chloride density gradient method (Ajuebor et al., 2018). After concentration, the phage suspension was dropped on a carbon-coated grid and left to be absorbed for 15 min. The sample was then stained with 2% (w/v) uranyl acetate in the dark for 10 min and observed under an H-7650 TME (Hitachi Limited, Tokyo, Japan) equipped with a CDD camera at an accelerating voltage of 100 kV.

#### Phage Titer and Optimal Multiplicity of Infection

The phage titer was determined through the double-layer agar method after serial dilution of the phage stock solution and calculated using the following formula: PFU/ml = number of plaques × dilution factor × 10. The multiplicity of infection (MOI) was expressed as the ratio of phages to host bacterial counts during infection. First, logarithmic-growth phase host bacteria at a concentration of 10^6^ colony-forming units (CFUs)/ml [Optical density at 600 nm (OD_600 *nm*_) = 0.18] were prepared by the correlation curve between the OD_600 *nm*_ and CFUs. Then, 100 μl of the diluted phages (MOI = 0.0001, 0.001, 0.01, 0.1, 1, 10, and 100) was mixed with 100 μl of host bacteria (10^6^ CFU/ml) at 37°C for 10 min. Subsequently, 5 ml of LB medium (2 mM CaCl_2_) was added to the mixture. After incubation at 37°C for 10 h, the precipitate was removed through centrifugation at 12,000 × *g* for 5 min. Then, the supernatant was filtered using a 0.45-μm syringe filter. The double-layer agar method was performed to determine the phage titer, with the highest titer of infection being considered the optimal MOI (Li et al., 2019; Luo et al., 2021). Each MOI test was performed based on three parallel experiments.

#### One-Step Growth Curve

The one-step growth curve was determined according to a previous report with minor modifications (Bao et al., 2011). The host strain *S*. Albany was cultured in 5 ml of LB medium at 37°C to an OD_600 *nm*_ of approximately 0.2. Then, the bacterial suspension was further diluted to 10^7^ CFU/ml. Thereafter, 1 ml of the bacterial culture was centrifuged at 12,000 × *g* for 2 min and resuspended with 1 ml of a 10^5^-PFU/ml phage suspension to achieve an MOI of 0.01. The mixture was incubated at 37°C for 15 min and centrifuged at 12,000 × *g* for 2 min. After that, the pellet was resuspended in 10 ml of LB medium (2 mM CaCl_2_). The mixture was immediately incubated at 37°C with shaking at 200 rpm. During the 100-min incubation, 100 μl of the sample was collected at 5-min intervals to determine the phage titer using the double-layer agar method. The one-step growth curve was described based on the correlation between the phage titer logarithm (log_10_ PFU/ml) and the infection time. This experiment was carried out in triplicate. The latent period and burst size of the phages could be determined through the one-step growth curve. The latent period was the time interval between the absorption and the beginning first burst. The burst size was the ratio of the final number of increased phages to the initial number of bacteria.

#### Stability Studies

The phages were studied for their ability to survive at different temperatures and pH values. For the thermal stability test, the phage suspension (10^8^ PFU/ml) was incubated at 4°C, 20°C, 30°C, 37°C, 40°C, 50°C, 60°C, 70°C, and 80°C and collected after 1 h. For the pH stability test, the phage suspension was added to LB medium with different pH values (pH was adjusted from 2 to 13 using HCl or NaOH) to a final concentration of 10^8^ PFU/ml. Subsequently, the culture was incubated at 37°C for 1 h. After the respective heat and pH experiments, the phage titer was also determined using the double-layer agar method.

#### Genomic DNA Extraction, High-Throughput Sequencing, and Bioinformatics Analysis

The phage suspension was first treated with RNase (3 μg/ml) and DNase I (0.1 U/μl) at 37°C for 1 h to degrade the bacterial nucleic acids. After thermal inactivation, 50 μg/ml of proteinase K, 20 mM ethylene diamine tetraacetic acid (EDTA), and 0.5% (v/v) sodium dodecyl sulfate (SDS) were mixed with the solution and incubated at 65°C for 30 min. Next, the DNA was extracted using saturated phenol, and the aqueous phase was collected after centrifugation at 12,000 × *g* for 5 min. An equal volume of phenol–chloroform–isoamyl alcohol (25:24:1) was added, completely mixed, and centrifuged at 12,000 × *g* for 5 min. This step was repeated twice. The supernatant was collected into a new centrifuge tube, mixed with an equal volume of isopropanol, and then maintained at −20°C for 30 min. After centrifugation at 12,000 × *g* and 4°C for 30 min, pellets were washed with 75% ethanol three times, then dissolved in 50 μl of deionized water, and stored at −20°C before use (Chun et al., 2020; Yang et al., 2020).

The DNA library was constructed using the QIAseq^TM^ FX DNA Library Kit (Qiagen, Hilden, Germany), and high-throughput sequencing was carried out using an Illumina HiSeq^TM^ 2500 (Illumina, San Diego, CA, United States) according to the manufacturer’s instructions. Low-quality reads were removed, and clean reads were assembled through the SPAdes v3.6.2 program (Bankevich et al., 2012). The whole-genome sequence was searched against other nucleotide/protein sequences using the BLAST program of the National Center for Biotechnology Information (NCBI). After that, some similar phage genomes within *Itachcavirus* were downloaded from the NCBI database to assess the average nucleotide identity (ANI) using the ANIb (average nucleotide identity based on BLAST calculation) tool in the pyani package. Coregene 3.5 program^1^ (Mahadevan et al., 2009a,b; Mahadevan and Seto, 2010) was conducted for comparative analysis of phages at the protein level. Open reading frames (ORFs) that might encode gene products were annotated using Prokka 1.13.7 (Seemann, 2014). Then, the putative functions of ORFs were manually searched using BLASTp against the Non-Redundant Protein Database of NCBI, where the score was set to >50%. In addition, pairwise genome comparison was done by BLASTn and illustrated using the Easyfig Tool 2.2.3 (Sullivan et al., 2011). The phylogenetic tree was determined based on the major capsid protein (Supplementary Table 3), using MEGA-X (v.10.0.5) (Kumar et al., 2018) via the neighbor-joining method with 1,000 bootstrap replicates. The antimicrobial resistance genes and virulence factors were screened in the Comprehensive Antibiotic Resistance Database (CARD) (Jia et al., 2017) and Virulence Factor Database (VFDB) (Chen et al., 2016), respectively.

### Lytic Activity of Phages

The lytic activity of phages against *Salmonella* was examined *in vitro*. *S.* Albany, which carriers nine drug-resistance genes [*aac(6’)-Iaa_1*, *mdf(A)_1*, *sul1_10*, *floR_2*, *tet(G)_2*, *dfrA1_8*, *ant(3”)-Ia_1*, *blaCARB-2_1*, and *sul1_5*], was used as the host strain. First, 100 μl of logarithmic-growth phase host bacterial culture (10^6^ CFU/ml) was mixed with an equal volume of phage suspension (10^8^–10^2^ PFU/ml) in a 96-well microplate to achieve the different MOIs of 0.0001, 0.001, 0.01, 0.1, 1, 10, and 100, followed by incubation at 37°C for 24 h. The OD_600 *nm*_ was measured at 0.5-h intervals. An *S.* Albany culture without phages was used as a positive control, whereas phages mixed with LB medium served as a negative control. The independent experiment was performed based on three replicates. The values of OD_600 *nm*_ were measured using an ELISA microplate reader (BioTek Instruments Inc., Winooski, VT, United States).

### Food Application

Two different types of food, including pasteurized milk and chicken meat, were purchased from local supermarkets in Guangzhou and stored at 4°C for only one night for immediate use. For the liquid sample, 19.6 ml of pasteurized milk was inoculated with 200 μl of *S.* Albany at 10^8^ CFU/ml and then treated with 200 μl of phage solution (10^6^ PFU/ml) or PBS buffer. Each sample was incubated at 37°C with shaking at 200 rpm. At 0, 2, 4, 6, 8, 10, 12, and 24 h, 500 μl of the homogenate was quickly centrifuged at 5,000 × *g* for 5 min to collect bacteria, which were resuspended in PBS. Samples were 10-fold diluted in 4.5 ml of PBS, and 100 μl of an appropriate dilution was plated on chromogenic *Salmonella* agar (HKM, Guangzhou, China) to enumerate the viable counts of *Salmonella* colonies (typical purple-colored colonies). The chicken meat was cut into 3 cm × 3 cm × 1 cm pieces using a sharp knife. Both sides of the chicken meat piece were washed with 70% ethanol and placed in the center of sterile Petri dishes, which were treated with UV for at least 1 h to eliminate indigenous bacteria and phages. Thereafter, 100 μl of *S.* Albany at 10^8^ CFU/mL was applied onto the surface of each sample (10 spots and one spot volume was approximately 10 μl). After air drying in a safety cabinet for 15 min to allow the attachment of bacteria, 100 μl of phage solution (10^6^ PFU/ml) was spotted in the same place and then stored at 37°C for 2, 4, 6, 8, 10, 12, and 24 h. An equal volume of PBS buffer was used instead of phages as a control. Each sample was withdrawn at the indicated time points and homogenized with 4.5 ml of PBS buffer for 2 min. The bacteria from phage-treated samples were also recovered through centrifugation at 5,000 × *g* for 5 min. *Salmonella* numbers were determined in a manner similar to that applied for the milk samples. Each condition was tested in duplicate.

### Statistical Analysis

Results were expressed as mean values ± standard deviations. GraphPad Prism 8.0.1 was used for statistical analysis. Two-way ANOVA with multiple comparisons was performed to determine differences between groups. A *p-*value less than 0.05 was considered statistically significant in all cases.

## Results

### Phage Isolation and Their Host Range

A total of five *Salmonella* phages were isolated from soil samples in this study. We tested the host spectrum of these phages against the 24 most prevalent *Salmonella* serovars. We accordingly found that phage vB_Sal_TR1, vB_SalP_TR2, vB_Sal_TR3, vB_Sal_TR4, and vB_Sal_TR5 were able to lyse 54.2% (13/24), 20.8% (5/24), 87.5% (21/24), 4.2% (1/24), and 16.7% (4/24) of tested *Salmonella* serovars, respectively. Phage vB_SalP_TR2 was shown to be a novel phage with high lytic potential against the *Salmonella* serovars Albany, Corvallis, Newport, Kottbus, and Istanbul (Table 1). In addition, this phage has not been reported by other researchers. Based on these results, we selected phage vB_SalP_TR2 for further experiments.

### Morphological Analysis

**FIGURE 1:**
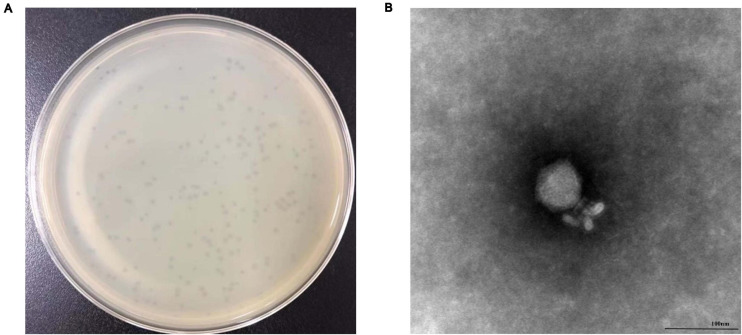
Morphology of *Salmonella* phage vB_SalP_TR2. **(A)** Phage plaques formed on double-layer agar plates. **(B)** Transmission electron microscopy image of vB_SalP_TR2. Phage vB_SalP_TR2 produced clear plaques and belongs to the *Podoviridae* family.

We observed that phage vB_SalP_TR2 formed round plaques of approximately 2 mm on the double-layer agar plate (Figure 1A). Our obtained TEM image revealed that phage vB_SalP_TR2 harbored an icosahedral head, approximately 62 ± 0.5 nm in diameter and 60 ± 1 nm in length, and a short tail, 35 ± 1 nm in length (Figure 1B). The morphological characteristics of phage vB_SalP_TR2 suggested that it belongs to the *Podoviridae* family of the *Caudovirales* order.

### Optimal Multiplicity of Infections and Latent Period

**FIGURE 2:**
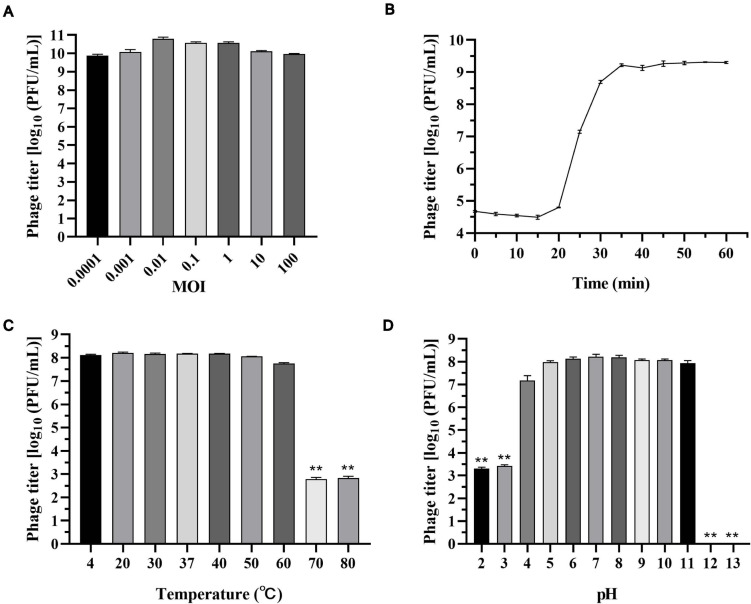
Biological characteristics of *Salmonella* phage vB_SalP_TR2. **(A)** Phage titers were measured at different multiplicity of infection (MOIs) (0.0001 to 100). **(B)** The one-step growth curve of vB_SalP_TR2 using *S.* Albany as the host in LB medium at MOI of 0.01. **(C)** Temperature stability. Phages were incubated at different temperatures (4°C, 20°C, 30°C, 37°C, 40°C, 50°C, 60°C, 70°C, and 80°C) for 1 h. **(D)** pH stability. Phages were incubated at different pHs (2, 3, 4, 5, 6, 7, 8, 9, 10, 11, 12, and 13) for 1 h. Data are expressed as the mean ± SD. **Significant at *p* < 0.05.

As shown in Figure 2A, phage titers were similar at different MOIs, indicating that the phage titer of vB_SalP_TR2 was less affected by MOI. We chose MOI = 0.01, at which the phage reached its relatively high titer of 10.8 log_10_ PFU/ml for performing the following experiments. The one-step growth curve of vB_SalP_TR2 propagated on host *S.* Albany in LB medium revealed that the latent and rise periods were approximately 15 and 20 min (Figure 2B), respectively. The average burst size was estimated to be 211 PFU/cell.

### Thermal and pH Stability

The thermal stability test showed that phage vB_SalP_TR2 was relatively stable at temperatures from 4°C to 60°C and more sensitive to high temperatures. The averaged titer was 8.1 log_10_ PFU/ml upon exposure at 4°C, 20°C, 30°C, 37°C, 40°C, 50°C, or 60°C for 1 h, and no statistical differences (*p* > 0.05) were noted among them. However, after incubation at 70 and 80°C for 1 h, we found that the survival rate declined to approximately 2.8 log_10_ PFU/ml (*p* < 0.05) (Figure 2C). In addition, we noticed that phage vB_SalP_TR2 retained a high titer (>7 log_10_ PFU/ml) from pH 4 to pH 11 for at least 1 h. However, it showed a significant decrease (*p* < 0.05) in titer at pH 2 and 3, with phage titers of only 3.3 and 3.4 log_10_ PFU/ml, respectively. At pH 12 and 13, we observed that nearly no phages survived the strong alkaline environment (*p* < 0.05) (Figure 2D).

### General Features of the Phage Genome

We found that phage vB_SalP_TR2 has a linear dsDNA genome consisting of 71,453 bp with a G + C content of 40.64%, which is lower than that of its host *Salmonella* strains (52.10%). Bioinformatics analysis revealed that phage vB_SalP_TR2 contained 96 ORFs and six tRNAs, and its termini was a short exact direct repeat end. More specifically, we identified that 35 (36.5%) of the 96 putative ORFs were assigned to functional genes (Supplementary Table 1), whereas the remaining 61 (63.5%) were annotated as hypothetical proteins. The predicted gene products were divided into five modules, DNA metabolism (DNA replication and encapsulation), structure, packaging, host lysis, and additional functions. Moreover, we did not detect any virulence and antibiotic resistance genes in the genome of phage vB_SalP_TR2, indicating that this phage is biologically safe for practical use.

**FIGURE 3:**
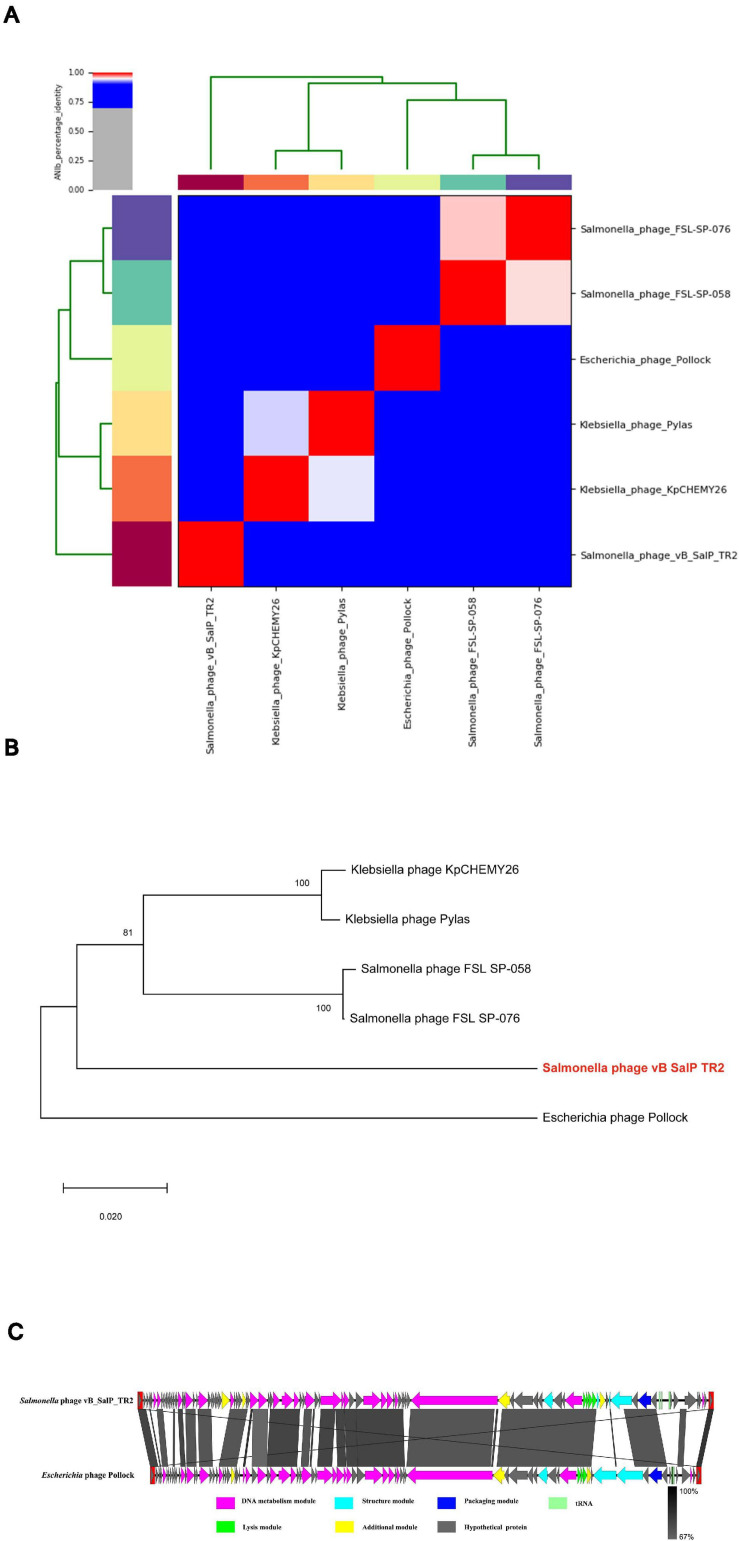
Analysis of *Salmonella* phage vB_SalP_TR2 genome. **(A)** Heatmap of the average nucleotide identity (ANI) values among vB_SalP_TR2 and five related phages within *Itachcavirus* genus. ANIb tool in the pyani package is used. ANI values ranging from 0 (0%) to 1 (100%) were generated according to a matrix of percentage identity. **(B)** Phylogenetic tree of the major capsid protein sequences showing the phylogenetic position of phage vB_SalP_TR2 (in red and bold) within the *Itachcavirus* genus. The phylogenetic tree was generated in MEGA-X (v.10.0.5) using neighbor-joining method with *p*-distance values and bootstrap replicate of 1,000. Consensus support (%) >50% is marked at each node. Scale bar represents 0.02 substitutions. **(C)** Genomic comparison of vB_SalP_TR2 and *Escherichia* phage Pollock generated using Easyfig. Genes with different functions are denoted by different colors. The similarities are displayed as gray lines and the level of identification is indicated by the gray shading.

An online BLASTn search revealed that the genomic sequence of phage vB_SalP_TR2 is most closely related to *Salmonella* phage FSL SP-076 (61% query coverage, 77.70% identified, accession number NC_021782.1) and FSL SP-058 (63% query coverage, 80.60% identified, accession number NC_021772.1) (Moreno Switt et al., 2013), *Klebsiella* phage Pylas (45% query coverage, 77.08% identified, accession number NC_049444.1) (Powell et al., 2019) and KpCHEMY26 (49% query coverage, 76.87% identified, accession number NC_049467.1) (Yerushalmy et al., 2019), and *Escherichia* phage Pollock (42% query coverage, 76.61% identified, accession number NC_027381.1) (Patel et al., 2015). Upon analyzing the results of the heatmap (Figure 3A) and regarding the ANIb percentage identification data (Supplementary Table 2), phage vB_SalP_TR2 displayed <75% nucleotide similarity to other phages within the *Itachcavirus* genus, *Podoviridae* family (phages FSL SP-076, FSL SP-058, Pylas, KpCHEMY26, and Pollock). At the protein level, computational analysis of CoreGenes showed that phage vB_SalP_TR2 shared 75 (91.5%), 76 (88.4%), 75 (81.5%), 70 (86.4%), and 69 (81.2%) homologs with phages FSL SP-076, FSL SP-058, Pylas, KpCHEMY26, Pollock, respectively. In addition, phage vB_SalP_TR2 possessed capsid protein with 81% amino acid similarity to the known *Salmonella* and *Klebsiella* phages (Figure 3B). Combined, these results indicated that phage vB_SalP_TR2 is a new species of *Itachcavirus* genus. The global genome comparison map revealed differences between vB_SalP_TR2 and *Escherichia* phage Pollock (Figure 3C). There were some ORFs with low sequence identities among them, and there were also some region arrangements in phage vB_SalP_TR2 relative to the *Escherichia* phage Pollock. These results also suggest that phage vB_SalP_TR2 is a novel *Salmonella* phage. The complete genome sequence was deposited into the GenBank database with the accession number MW544066.1.

### Bacteriolytic Activity of Phages

**FIGURE 4:**
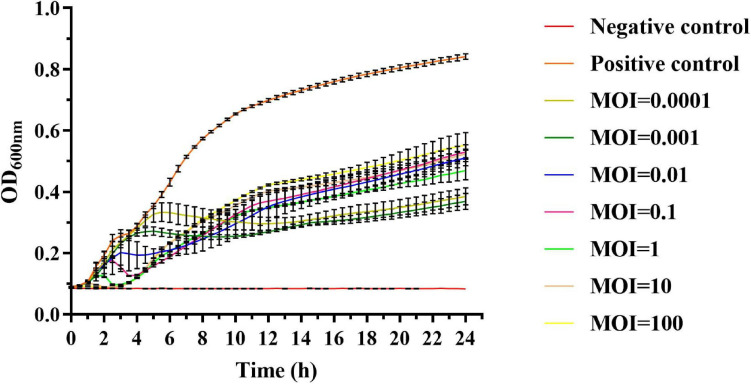
*In vitro* lytic activity of phage vB_SalP_TR2 against *S.* Albany in Luria–Bertani (LB) medium with different MOIs (0.0001 to 100) at 37°C. OD_600 *nm*_ was measured at 30-min intervals. Non-phage-infected bacterial culture was the positive control, and LB medium was the negative control. Error bars indicate the standard deviation.

We then tested if phage vB_SalP_TR2 could inhibit the dynamic growth of *Salmonella*. Accordingly, we observed that without treatment with phage vB_SalP_TR2, *S.* Albany reached its logarithmic phase at 1 h and sharply increased after 1 h, whereas the OD_600 *nm*_ values of the negative control containing LB medium alone remained unchanged. In contrast, following treatment with phage vB_SalP_TR2 at different MOIs (MOI = 0.0001, 0.001, 0.01, 0.1, 1, 10, and 100, respectively), the OD_600 *nm*_ values were found to always be less than that of the positive control (*p* < 0.05), indicating that phage vB_SalP_TR2 could efficiently inhibit bacterial growth. There were no obvious differences in bacterial inhibition among the various MOIs (Figure 4). The inhibitory effect at MOI = 0.01 and 0.001 was better, but not at MOI = 0.1, and the possible reason might be that the measurement volume was small (200 μl), and the culture condition was static. In addition, this strain, *S.* Albany, carries a certain number of drug-resistance genes, suggesting that this phage might have the potential to inhibit drug-resistant strains.

### Efficiency of Phages in Reducing *Salmonella* Numbers in Pasteurized Milk and Chicken Meat

**FIGURE 5:**
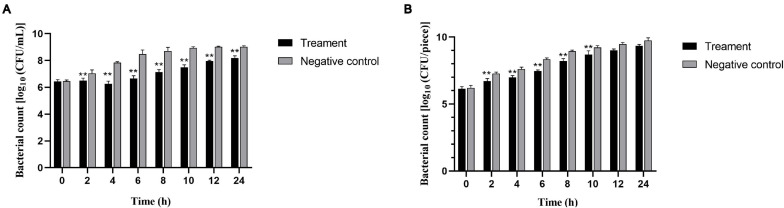
Effectiveness of *Salmonella* phage vB_SalP_TR2 in reducing *S.* Albany in milk **(A)** and chicken meat **(B)**. The phage vB_SalP_TR2 and *S.* Albany were mixed at an MOI of 0.01 in milk and chicken meat. The number of *S.* Albany was determined at 2, 4, 6, 8, 10, 12, and 24 h. All data are shown as the mean ± SD. **Significant at *p* < 0.05.

We also evaluated the ability of phage vB_SalP_TR2 to reduce *S.* Albany contamination in pasteurized milk and chicken meat at different time points after storage at 37°C. In the milk assay, the phage-treated group resulted in a significant reduction in the viable counts of the inoculated bacteria compared with those of the phage-free controls (*p* < 0.05). The viable counts subsequently recovered slightly after 4 h of treatment. The maximum reduction for *S.* Albany was demonstrated to be 1.8 log_10_ CFU/ml at 6 h (Figure 5A). In the meat assay, the application of phage vB_SalP_TR2 to the surface of *S.* Albany artificially contaminated chicken meat resulted in a significant reduction in bacterial growth compared with those of the controls (*p* < 0.05) after 2, 4, 6, 8, and 10 h of treatment at 37°C, with a maximum reduction of 0.9 log_10_ CFU/piece at 6 h. However, we noted that the reduction in the numbers of *S.* Albany was not statistically significant (*p* > 0.05) after incubation at 12 and 24 h (Figure 5B). In general, phage vB_SalP_TR2 was much more effective against *Salmonella* in pasteurized milk than in chicken meat.

## Discussion and Conclusion

Phages are ubiquitous in the environment and particularly enriched in the soil, with numbers reaching as high as 10^10^/g of dry weight (Williamson et al., 2017). In the food industry, to control pathogenic bacteria, phages have been widely used as biological control agents. For example, several major *Salmonella* phage products, including SalmoFresh^TM^ (Intralytix, Inc., Baltimore, United States), Salmonelex^TM^ (Micreos BV, Wageningen, Netherlands), and BioTector^®^ (CheilJedang Corporation, Seoul, South Korea), have been widely used in meat products (Goodridge et al., 2018; LeLièvre et al., 2019; Lewis and Hill, 2020). However, the major limitation in using the phages as biological control for *Salmonella* is the narrow host range since most isolated phages are usually specific to *S. enteritidis* or *S. typhimurium*. Although genetic engineering could be employed as a new approach to widen the host range, this would require advanced technology (Lin et al., 2012; Kilcher and Loessner, 2019). Therefore, finding more *Salmonella* phages focused on different serovars with high lytic capacity is still needed. In our study, five *Salmonella* phages were isolated from soil samples using 24 common serovars as hosts. Phage vB_SalP_TR2 was found to be a novel phage, successfully infecting *S.* Albany, *S.* Corvallis, *S.* Newport, *S.* Kottbus, and *S.* Istanbul. In the one-step growth curve result, the latent period of phage vB_SalP_TR2 was 15 min, and the average burst size was 211 PFU/cell. The latent period of phage vB_SalP_TR2 is shorter than that (25–65 min) of many other reported *Salmonella* phages (Huang et al., 2018; El-Dougdoug et al., 2019; Duc et al., 2020). The shorter latent period has been positively related to effectively inactivating bacteria (Li et al., 2020), indicating the utility of phage vB_SalP_TR2 in the biocontrol of *Salmonella* in practical settings. Resistance to heat and pH is essential for biocontrol applications. Phage vB_SalP_TR2 exhibited relatively high thermal stability as the phage titer could be detected after exposure to 80°C for 1 h, implying its compatibility with pasteurization in practical food processing. Phage vB_SalP_TR2 was also found to retain a high activity with a wide pH range, allowing for its utilization in food matrices with different pH values. For example, fruits and meat generally have a low pH (pH 4∼6), whereas milk has a neutral pH.

Whole-genome sequencing has been increasingly recognized as a necessary condition to ensure the safety of phage preparation. Based on the study of phylogeny and morphology, phage vB_SalP_TR2 was identified as a new member of the *Itachcavirus* genus, *Podoviridae* family. Genomic analysis showed that phage vB_SalP_TR2 did not contain any genes related to virulence and antibiotic resistance genes, making the phages suitable for food applications.

The efficiency of phage vB_SalP_TR2 in controlling *Salmonella* contamination in fresh food was evaluated in pasteurized milk and chicken meat; these products were used to simulate liquid and solid food ingredients. Phage vB_SalP_TR2 could effectively inhibit the growth of *S.* Albany, with its performance in milk surpassing that in chicken meat. It is believed that this phenomenon is caused by the inability of the phages to move and reach the host on the surface of the solid food (Guenther et al., 2012; Duc et al., 2020). The previously reported bacteriophages (PA13076 and LPST10) are useful in reducing the number of *Salmonella* in various food ingredients. Phage vB_SalP_TR2 was found to exhibit greater efficiency than phage PA13076, which could decrease *S. enteritidis* ATCC 13,076 counts by 1 log CFU/ml in milk (Bao et al., 2015). Wang et al., demonstrated that phage LPST10 causes the vitality of *S. typhimurium* ATCC 14,028 to decrease by 1.1 log CFU/sample (2-cm diameter and 1-cm thickness) on packaged sausage at 28°C (Huang et al., 2018). Similar to previous studies on phage-based biocontrol, bacterial regrowth was also observed during bacteriolytic experiment, likely due to the emergence of bacterial resistance mechanisms, including restricted modification systems, spontaneous mutations, and adaptive immunity through the CRISPR-Cas system (Oechslin, 2018). Several solutions have been proposed to delay bacterial regeneration, such as the use of high concentrations of phage suspensions (Guenther et al., 2009) and the use of phage cocktails or phages in combination with other antibacterial agents to minimize the impact (Abhisingha et al., 2020; Alves et al., 2020; Wong et al., 2020). In practice, food is naturally contaminated with a very low pathogen titer (Zhu et al., 2014). Since they are not usually exposed to inappropriate environments for a long time, bacterial hosts could be eliminated before they could develop resistance to phages (Gurney et al., 2020).

However, one limitation of the current study was that we could not identify the mechanism underlying the ability of phage vB_SalP_TR2 to lyse *Salmonella* partial serovars. Further work will be conducted to investigate the recognition and lysis mechanism, and the interaction between the phages and its hosts, and to characterize other phages.

In summary, a novel *Salmonella* phage vB_SalP_TR2 was isolated. Phage vB_SalP_TR2 exhibited superior anti-*Salmonella* activities, relative high thermo- and acid tolerance, and biological safety, showing great potentials in the control of *Salmonella* in a food-processing environment.

## Data Availability Statement

The datasets presented in this study can be found in online repositories. The names of the repository/repositories and accession number(s) can be found below: https://www.ncbi.nlm.nih.gov/, MN544066.

## Author Contributions

JZ, QY, and YS conceived and designed the experiments. YS and QS performed the experiment and drafted the manuscript. HC, SY, MD, and FZ contributed to the data analysis. QW and MC reviewed the manuscript. All authors read and approved the submission of the final version of the manuscript for publication.

## Conflict of Interest

The authors declare that the research was conducted in the absence of any commercial or financial relationships that could be construed as a potential conflict of interest.

## Footnotes

1 http://binf.gmu.edu:8080/CoreGenes3.5/BatchCoreGenes.html
