# Supplementary material for: Isolation and Characterization of a Novel Salmonella Phage vB_SalP_TR2
Source: Front Microbiol. 2021 Jun 21;12:664810. doi: 10.3389/fmicb.2021.664810 (PMC8256156; doi:10.3389/fmicb.2021.664810)
Supplement: Supplementary file 1 [file Table_1.DOCX]

Table S1 Predicted protein function of of *Salmonella* phage vB_SalP_TR2.

| ORFs | Putative function | E vaule | Identity | Best phage homolog (Protein accession number) |
| --- | --- | --- | --- | --- |
| gp7 | GTP binding protein | 1.00E-53 | 69.75% | *Salmonella* phage FSL SP-058 (YP_008239411.1) |
| gp8 | DNA binding protein | 2.00E-49 | 61.90% | *Klebsiella* phage Pylas (YP_009881984.1) |
| gp19 | ADP-ribose-binding protein | 2.00E-95 | 77.84% | *Klebsiella* phage KpCHEMY26 (YP_009884209.1) |
| gp22 | RNA polymerase subunit | 2.00E-178 | 77.20% | *Salmonella* phage FSL SP-076 ([YP_008240153.1](https://www.ncbi.nlm.nih.gov/protein/YP_008240153.1?report=genbank&log$=prottop&blast_rank=8&RID=7UY91JH9013)) |
| gp25 | RNA polymerase | 0.00E+00 | 71.15% | *Escherichia* phage Pollock (YP_009152120.1) |
| gp32 | agglutinating adhesin | 1.00E-156 | 72.05% | *Salmonella* phage FSL SP-058 (YP_008239431.1) |
| gp33 | deoxyuridine 5'-triphosphate | 1.00E-89 | 87.25% | *Salmonella* phage FSL SP-076 (YP_008240162.1) |
| gp37 | tellurite resistance TerB family protein | 6.00E-86 | 92.62% | *Escherichia* phage Pollock (YP_009152128.1) |
| gp40 | NTPase | 6.00E-171 | 69.82% | *Klebsiella* phage KpCHEMY26 (YP_009884194.1) |
| gp41 | metallopeptidase | 3.00E-175 | 63.11% | *Escherichia* phage Pollock (YP_009152135.1) |
| gp44 | CLP protease | 2.00E-178 | 88.89% | *Klebsiella* phage KpCHEMY26 (YP_009884190.1) |
| gp45 | metalloprotease | 0.00E+00 | 88.10% | *Klebsiella* phage KpCHEMY26 (YP_009884188.1) |
| gp46 | serine/threonine-protein phosphatase | 5.00E-107 | 65.04% | *Klebsiella* phage Pylas ([YP_009882018.1](https://www.ncbi.nlm.nih.gov/protein/YP_009882018.1?report=genbank&log$=prottop&blast_rank=9&RID=7UVDHZ7J013)) |
| gp49 | ATP-dependent DNA helicase | 0.00E+00 | 69.10% | *Salmonella* phage FSL SP-058 (YP_008239448.1) |
| gp52 | DNA polymerase | 0.00E+00 | 82.97% | *Klebsiella* phage KpCHEMY26 (YP_009884181.1) |
| gp53 | HNH endonuclease | 2.00E-56 | 89.80% | *Klebsiella* phage KpCHEMY26 (YP_009884180.1) |
| gp54 | FAD-dependent thymidylate synthase | 2.00E-151 | 95.73% | *Klebsiella* phage Pylas (YP_009882028.1) |
| gp57 | DNA primase | 0.00E+00 | 85.83% | *Klebsiella* phage KpCHEMY26 (YP_009884176.1) |
| gp58 | AAA family ATPase | 4.00E-151 | 86.36% | *Escherichia* phage Pollock (YP_009152153.1) |
| gp59 | single-strand DNA binding protein | 1.00E-109 | 70.45% | *Salmonella* phage FSL SP-076 (YP_008240186.1) |
| gp61 | holliday junction resolvase | 8.00E-70 | 80.31% | *Klebsiella* phage KpCHEMY26 (YP_009884172.1) |
| gp67 | virion DNA-directed RNA polymerase | 0.00E+00 | 73.66% | *Salmonella* phage FSL SP-058 (YP_008239463.1) |
| gp68 | acyl-CoA reductase | 8.00E-180 | 57.35% | *Escherichia* phage Pollock (YP_009152161.1) |
| gp73 | major capsid protein | 0.00E+00 | 87.37% | *Salmonella* phage FSL SP-076 (YP_008240197.1) |
| gp76 | chromosome partitioning protein | 0.00E+00 | 75.47% | *Klebsiella* phage Pylas (YP_009882049.1) |
| gp77 | antiholin/holin protein | 1.00E-58 | 72.58% | *Klebsiella* phage Pylas (YP_009882050.1) |
| gp78 | holin | 3.00E-21 | 56.10% | *Klebsiella* phage KpCHEMY26 (YP_009884156.1) |
| gp79 | lysozyme | 8.00E-89 | 79.14% | *Salmonella* phage FSL SP-058 (YP_008239475.1) |
| gp80 | lysis protein | 1.00E-88 | 75.29% | *Salmonella* phage FSL SP-058 (YP_008239476.1) |
| gp81 | putative head protein | 6.00E-12 | 61.90% | *Salmonella* phage FSL SP-076 (YP_008240206.1) |
| gp82 | protease | 5.00E-86 | 65.05% | *Escherichia* phage Bp4 (YP_009031975.1) |
| gp84 | tail spike protein | 1.00E-09 | 63.64% | *Salmonella* phage Mutine (YP_009879669.1) |
| gp85 | tail protein | 0.00E+00 | 78.25% | *Salmonella* phage SKML-39 (YP_007236096.1) |
| gp87 | terminase | 0.00E+00 | 82.52% | *Escherichia* phage Pollock (YP_009152179.1) |
| gp94 | transcriptional regulator | 4.00E-10 | 56.86% | *Klebsiella* phage Pylas (YP_009884142.1) |
