# Supplementary material for: Isolation and Characterization of a Novel Salmonella Phage vB_SalP_TR2
Source: Front Microbiol. 2021 Jun 21;12:664810. doi: 10.3389/fmicb.2021.664810 (PMC8256156; doi:10.3389/fmicb.2021.664810)
Supplement: Supplementary file 2 [file Table_2.DOCX]

Table S2 ANIb percentage identify data.

|  | *Escherichia*_phage_Pollock | *Klebsiella*_phage_KpCHEMY26 | *Klebsiella*_phage_Pylas | *Salmonella*_phage_FSL-SP-058 | *Salmonella*_phage_FSL-SP-076 | ***Salmonella*_phage_vB_SalP_TR2** |
| --- | --- | --- | --- | --- | --- | --- |
| *Escherichia*_phage_Pollock | 1.00 | 0.77 | 0.77 | 0.81 | 0.81 | 0.75 |
| *Klebsiella*_phage_KpCHEMY26 | 0.77 | 1.00 | 0.94 | 0.79 | 0.79 | 0.75 |
| *Klebsiella*_phage_Pylas | 0.77 | 0.94 | 1.00 | 0.79 | 0.79 | 0.75 |
| *Salmonella*_phage_FSL-SP-058 | 0.81 | 0.79 | 0.79 | 1.00 | 0.95 | 0.76 |
| *Salmonella*_phage_FSL-SP-076 | 0.81 | 0.78 | 0.79 | 0.96 | 1.00 | 0.76 |
| ***Salmonella*_phage_vB_SalP_TR2** | 0.75 | 0.75 | 0.75 | 0.76 | 0.76 | 1.00 |
