# Supplementary material for: Isolation and Characterization of a Novel Salmonella Phage vB_SalP_TR2
Source: Front Microbiol. 2021 Jun 21;12:664810. doi: 10.3389/fmicb.2021.664810 (PMC8256156; doi:10.3389/fmicb.2021.664810)
Supplement: Supplementary file 3 [file Table_3.DOCX]

Table S3 The phages used in the construction of phylogenetic tree.

| No. | Name | Accession number^*^ |
| --- | --- | --- |
| 1 | *Escherichia* phage Pollock | NC_027381.1 |
| 2 | *Klebsiella* phage KpCHEMY26 | NC_049467.1 |
| 3 | *Klebsiella* phage Pylas | NC_049444.1 |
| 4 | *Salmonella* phage FSL SP-076 | NC_021782.1 |
| 5 | *Salmonella* phage FSL SP-058 | NC_021772.1 |

^*^ All data were downloaded from NCBI database (https://www.ncbi.nlm.nih.gov/).
